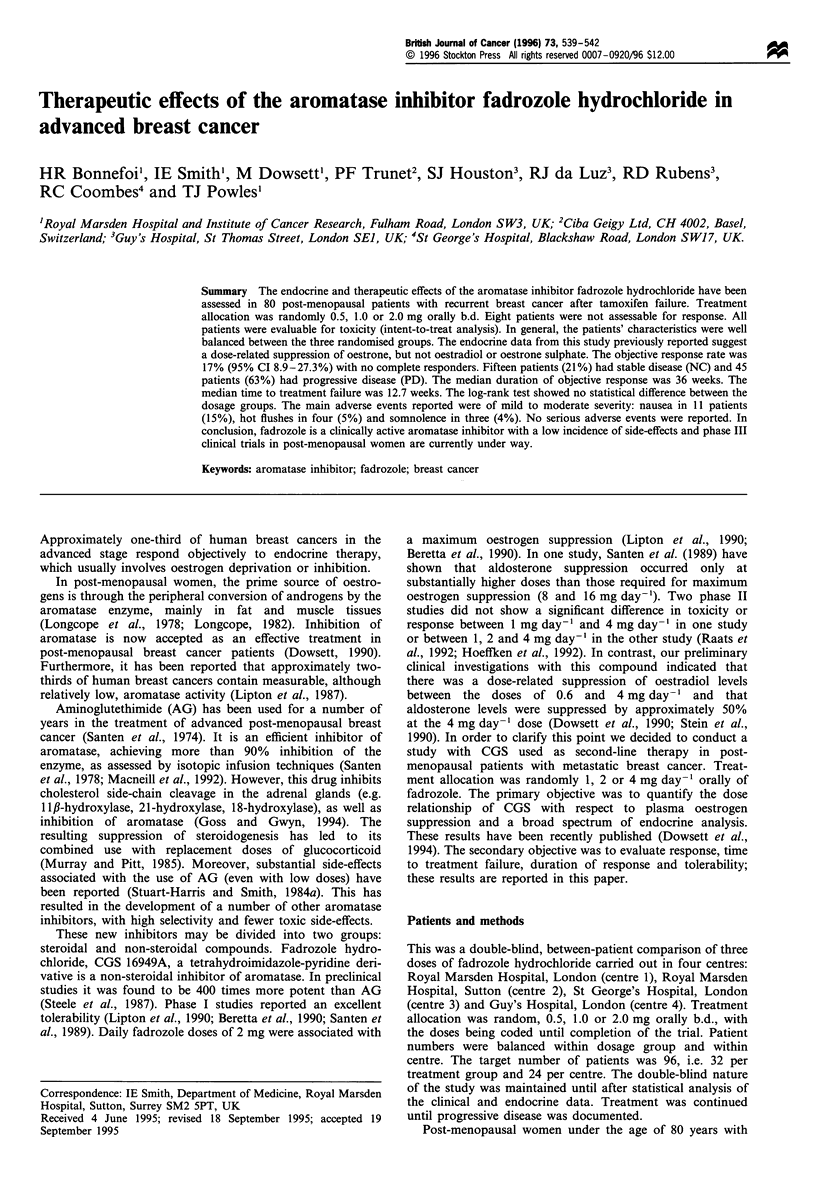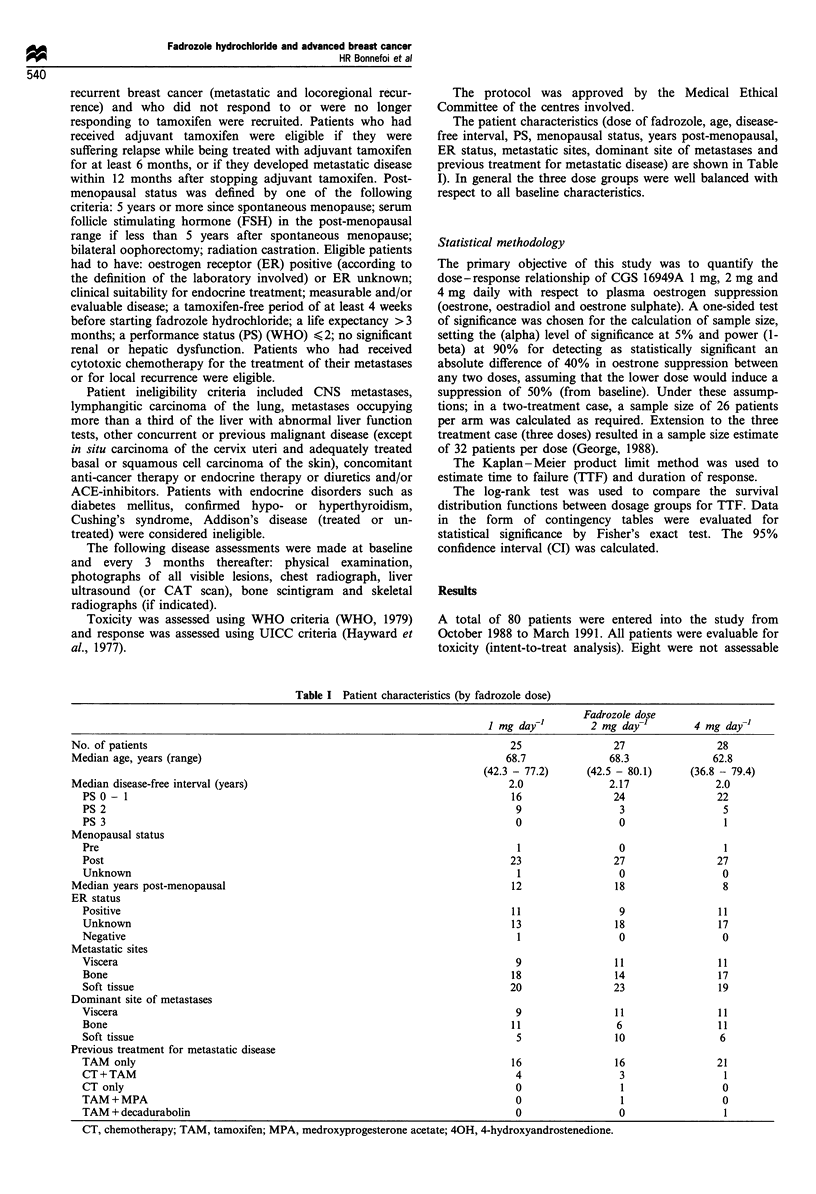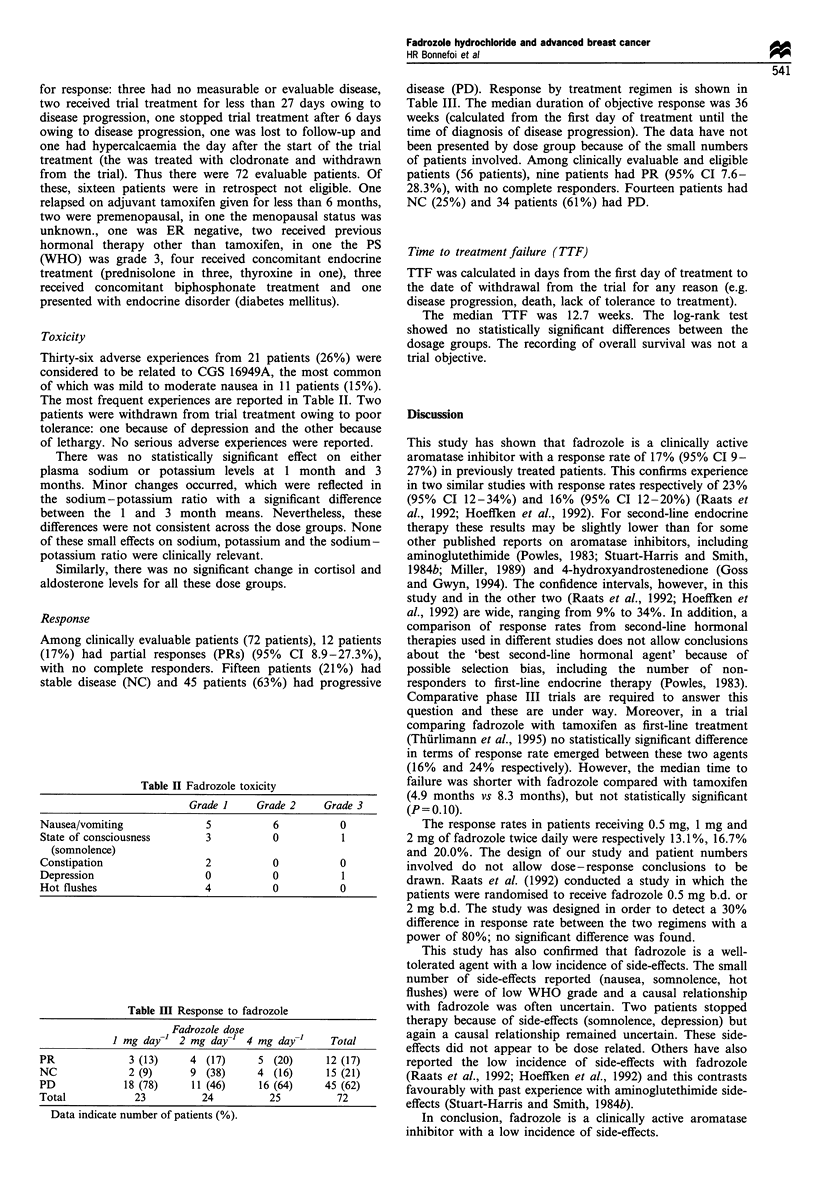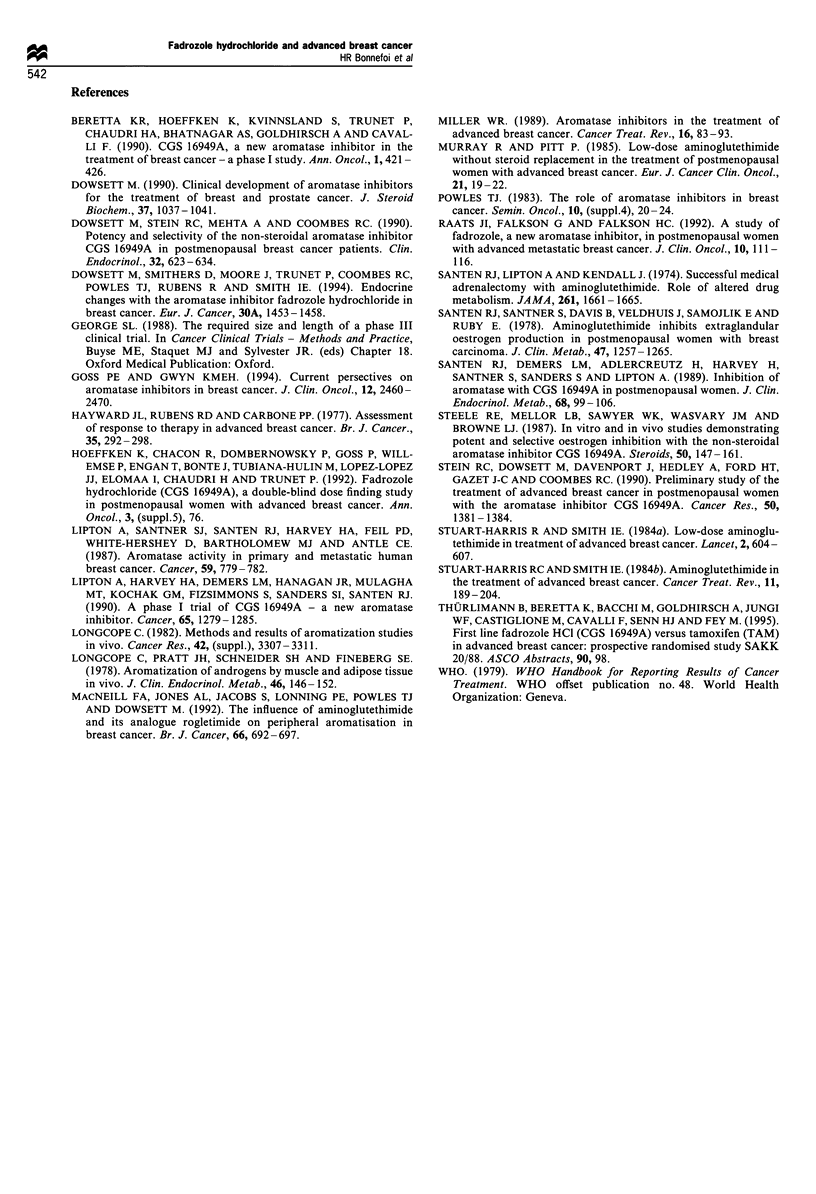# Therapeutic effects of the aromatase inhibitor fadrozole hydrochloride in advanced breast cancer.

**DOI:** 10.1038/bjc.1996.93

**Published:** 1996-02

**Authors:** H. R. Bonnefoi, I. E. Smith, M. Dowsett, P. F. Trunet, S. J. Houston, R. J. da Luz, R. D. Rubens, R. C. Coombes, T. J. Powles

**Affiliations:** Royal Marsden Hospital and Institute of Cancer Research, London, UK.

## Abstract

The endocrine and therapeutic effects of the aromatase inhibitor fadrozole hydrochloride have been assessed in 80 post-menopausal patients with recurrent breast cancer after tamoxifen failure. Treatment allocation was randomly 0.5, 1.0 or 2.0 mg orally b.d. Eight patients were not assessable for response. All patients were evaluated for toxicity (intent-to-treat analysis). In general, the patients' characteristics were well balanced between the three randomised groups. The endocrine data from this study previously reported suggest a dose-related suppression of oestrone, but not oestradiol or oestrone sulphate. The objective response rate was 17% (95% CI 8.9-27.3%) with no complete responders. Fifteen patients (21%) had stable disease (NC) and 45 patients (63%) had progressive disease (PD). The median duration of objective response was 36 weeks. The median time to treatment failure was 12.7 weeks. The log-rank test showed no statistical difference between the dosage groups. The main adverse events reported were mild to moderate severity: nausea in 11 patients (15%), hot flashes in four (5%) and somnolence in three (4%). No serious adverse events were reported. In conclusion, fadrozole is a clinically active aromatase inhibitor with a low incidence of side-effects and phase III clinical trials in post-menopausal women are currently under way.


British Journal of Cancer (1996) 73, 539-542
© 1996 Stockton Press All rights reserved 0007-0920/96 $12.00

### Therapeutic effects of the aromatase inhibitor fadrozole hydrochloride in advanced breast cancer

HR Bonnefoi[1], IE Smith[1], M Dowsett[1], PF Trunet[2], SJ Houston[3], RJ da Luz[3], RD Rubens[3], RC Coombes[4] and TJ Powles[1]

[1]*Royal Marsden Hospital and Institute of Cancer Research, Fulham Road, London SW3, UK;* [2]*Ciba Geigy Ltd, CH 4002, Basel, Switzerland;* [3]*Guy's Hospital, St Thomas Street, London SE1, UK;* [4]*St George's Hospital, Blackshaw Road, London SW17, UK.*

**Summary** The endocrine and therapeutic effects of the aromatase inhibitor fadrozole hydrochloride have been assessed in 80 post-menopausal patients with recurrent breast cancer after tamoxifen failure. Treatment allocation was randomly 0.5, 1.0 or 2.0 mg orally b.d. Eight patients were not assessable for response. All patients were evaluable for toxicity (intent-to-treat analysis). In general, the patients' characteristics were well balanced between the three randomised groups. The endocrine data from this study previously reported suggest a dose-related suppression of oestrone, but not oestradiol or oestrone sulphate. The objective response rate was 17% (95% CI 8.9-27.3%) with no complete responders. Fifteen patients (21%) had stable disease (NC) and 45 patients (63%) had progressive disease (PD). The median duration of objective response was 36 weeks. The median time to treatment failure was 12.7 weeks. The log-rank test showed no statistical difference between the dosage groups. The main adverse events reported were of mild to moderate severity: nausea in 11 patients (15%), hot flushes in four (5%) and somnolence in three (4%). No serious adverse events were reported. In conclusion, fadrozole is a clinically active aromatase inhibitor with a low incidence of side-effects and phase III clinical trials in post-menopausal women are currently under way.

**Keywords:** aromatase inhibitor; fadrozole; breast cancer

Approximately one-third of human breast cancers in the advanced stage respond objectively to endocrine therapy, which usually involves oestrogen deprivation or inhibition.

In post-menopausal women, the prime source of oestrogens is through the peripheral conversion of androgens by the aromatase enzyme, mainly in fat and muscle tissues (Longcope *et al.*, 1978; Longcope, 1982). Inhibition of aromatase is now accepted as an effective treatment in post-menopausal breast cancer patients (Dowsett, 1990). Furthermore, it has been reported that approximately two-thirds of human breast cancers contain measurable, although relatively low, aromatase activity (Lipton *et al.*, 1987).

Aminoglutethimide (AG) has been used for a number of years in the treatment of advanced post-menopausal breast cancer (Santen *et al.*, 1974). It is an efficient inhibitor of aromatase, achieving more than 90% inhibition of the enzyme, as assessed by isotopic infusion techniques (Santen *et al.*, 1978; Macneill *et al.*, 1992). However, this drug inhibits cholesterol side-chain cleavage in the adrenal glands (e.g. 11$\beta$-hydroxylase, 21-hydroxylase, 18-hydroxylase), as well as inhibition of aromatase (Goss and Gwyn, 1994). The resulting suppression of steroidogenesis has led to its combined use with replacement doses of glucocorticoid (Murray and Pitt, 1985). Moreover, substantial side-effects associated with the use of AG (even with low doses) have been reported (Stuart-Harris and Smith, 1984*a*). This has resulted in the development of a number of other aromatase inhibitors, with high selectivity and fewer toxic side-effects.

These new inhibitors may be divided into two groups: steroidal and non-steroidal compounds. Fadrozole hydrochloride, CGS 16949A, a tetrahydroimidazole-pyridine derivative is a non-steroidal inhibitor of aromatase. In preclinical studies it was found to be 400 times more potent than AG (Steele *et al.*, 1987). Phase I studies reported an excellent tolerability (Lipton *et al.*, 1990; Beretta *et al.*, 1990; Santen *et al.*, 1989). Daily fadrozole doses of 2 mg were associated with a maximum oestrogen suppression (Lipton *et al.*, 1990; Beretta *et al.*, 1990). In one study, Santen *et al.* (1989) have shown that aldosterone suppression occurred only at substantially higher doses than those required for maximum oestrogen suppression (8 and 16 mg day$^{-1}$). Two phase II studies did not show a significant difference in toxicity or response between 1 mg day$^{-1}$ and 4 mg day$^{-1}$ in one study or between 1, 2 and 4 mg day$^{-1}$ in the other study (Raats *et al.*, 1992; Hoeffken *et al.*, 1992). In contrast, our preliminary clinical investigations with this compound indicated that there was a dose-related suppression of oestradiol levels between the doses of 0.6 and 4 mg day$^{-1}$ and that aldosterone levels were suppressed by approximately 50% at the 4 mg day$^{-1}$ dose (Dowsett *et al.*, 1990; Stein *et al.*, 1990). In order to clarify this point we decided to conduct a study with CGS used as second-line therapy in post-menopausal patients with metastatic breast cancer. Treatment allocation was randomly 1, 2 or 4 mg day$^{-1}$ orally of fadrozole. The primary objective was to quantify the dose relationship of CGS with respect to plasma oestrogen suppression and a broad spectrum of endocrine analysis. These results have been recently published (Dowsett *et al.*, 1994). The secondary objective was to evaluate response, time to treatment failure, duration of response and tolerability; these results are reported in this paper.

### Patients and methods

This was a double-blind, between-patient comparison of three doses of fadrozole hydrochloride carried out in four centres: Royal Marsden Hospital, London (centre 1), Royal Marsden Hospital, Sutton (centre 2), St George's Hospital, London (centre 3) and Guy's Hospital, London (centre 4). Treatment allocation was random, 0.5, 1.0 or 2.0 mg orally b.d., with the doses being coded until completion of the trial. Patient numbers were balanced within dosage group and within centre. The target number of patients was 96, i.e. 32 per treatment group and 24 per centre. The double-blind nature of the study was maintained until after statistical analysis of the clinical and endocrine data. Treatment was continued until progressive disease was documented.

Post-menopausal women under the age of 80 years with

Correspondence: IE Smith, Department of Medicine, Royal Marsden Hospital, Sutton, Surrey SM2 5PT, UK
Received 4 June 1995; revised 18 September 1995; accepted 19 September 1995

recurrent breast cancer (metastatic and locoregional recurrence) and who did not respond to or were no longer responding to tamoxifen were recruited. Patients who had received adjuvant tamoxifen were eligible if they were suffering relapse while being treated with adjuvant tamoxifen for at least 6 months, or if they developed metastatic disease within 12 months after stopping adjuvant tamoxifen. Post-menopausal status was defined by one of the following criteria: 5 years or more since spontaneous menopause; serum follicle stimulating hormone (FSH) in the post-menopausal range if less than 5 years after spontaneous menopause; bilateral oophorectomy; radiation castration. Eligible patients had to have: oestrogen receptor (ER) positive (according to the definition of the laboratory involved) or ER unknown; clinical suitability for endocrine treatment; measurable and/or evaluable disease; a tamoxifen-free period of at least 4 weeks before starting fadrozole hydrochloride; a life expectancy >3 months; a performance status (PS) (WHO) ≤2; no significant renal or hepatic dysfunction. Patients who had received cytotoxic chemotherapy for the treatment of their metastases or for local recurrence were eligible.

Patient ineligibility criteria included CNS metastases, lymphangitic carcinoma of the lung, metastases occupying more than a third of the liver with abnormal liver function tests, other concurrent or previous malignant disease (except *in situ* carcinoma of the cervix uteri and adequately treated basal or squamous cell carcinoma of the skin), concomitant anti-cancer therapy or endocrine therapy or diuretics and/or ACE-inhibitors. Patients with endocrine disorders such as diabetes mellitus, confirmed hypo- or hyperthyroidism, Cushing's syndrome, Addison's disease (treated or untreated) were considered ineligible.

The following disease assessments were made at baseline and every 3 months thereafter: physical examination, photographs of all visible lesions, chest radiograph, liver ultrasound (or CAT scan), bone scintigram and skeletal radiographs (if indicated).

Toxicity was assessed using WHO criteria (WHO, 1979) and response was assessed using UICC criteria (Hayward *et al.*, 1977).

The protocol was approved by the Medical Ethical Committee of the centres involved.

The patient characteristics (dose of fadrozole, age, disease-free interval, PS, menopausal status, years post-menopausal, ER status, metastatic sites, dominant site of metastases and previous treatment for metastatic disease) are shown in Table I). In general the three dose groups were well balanced with respect to all baseline characteristics.

### *Statistical methodology*

The primary objective of this study was to quantify the dose–response relationship of CGS 16949A 1 mg, 2 mg and 4 mg daily with respect to plasma oestrogen suppression (oestrone, oestradiol and oestrone sulphate). A one-sided test of significance was chosen for the calculation of sample size, setting the (alpha) level of significance at 5% and power (1-beta) at 90% for detecting as statistically significant an absolute difference of 40% in oestrone suppression between any two doses, assuming that the lower dose would induce a suppression of 50% (from baseline). Under these assumptions; in a two-treatment case, a sample size of 26 patients per arm was calculated as required. Extension to the three treatment case (three doses) resulted in a sample size estimate of 32 patients per dose (George, 1988).

The Kaplan–Meier product limit method was used to estimate time to failure (TTF) and duration of response.

The log-rank test was used to compare the survival distribution functions between dosage groups for TTF. Data in the form of contingency tables were evaluated for statistical significance by Fisher's exact test. The 95% confidence interval (CI) was calculated.

### Results

A total of 80 patients were entered into the study from October 1988 to March 1991. All patients were evaluable for toxicity (intent-to-treat analysis). Eight were not assessable

**Table I** Patient characteristics (by fadrozole dose)

| | *Fadrozole dose* 1 mg $day^{-1}$ | 2 mg $day^{-1}$ | 4 mg $day^{-1}$ |
|---|---|---|---|
| No. of patients | 25 | 27 | 28 |
| Median age, years (range) | 68.7 (42.3 – 77.2) | 68.3 (42.5 – 80.1) | 62.8 (36.8 – 79.4) |
| Median disease-free interval (years) | 2.0 | 2.17 | 2.0 |
| PS 0 – 1 | 16 | 24 | 22 |
| PS 2 | 9 | 3 | 5 |
| PS 3 | 0 | 0 | 1 |
| Menopausal status | | | |
| Pre | 1 | 0 | 1 |
| Post | 23 | 27 | 27 |
| Unknown | 1 | 0 | 0 |
| Median years post-menopausal | 12 | 18 | 8 |
| ER status | | | |
| Positive | 11 | 9 | 11 |
| Unknown | 13 | 18 | 17 |
| Negative | 1 | 0 | 0 |
| Metastatic sites | | | |
| Viscera | 9 | 11 | 11 |
| Bone | 18 | 14 | 17 |
| Soft tissue | 20 | 23 | 19 |
| Dominant site of metastases | | | |
| Viscera | 9 | 11 | 11 |
| Bone | 11 | 6 | 11 |
| Soft tissue | 5 | 10 | 6 |
| Previous treatment for metastatic disease | | | |
| TAM only | 16 | 16 | 21 |
| CT + TAM | 4 | 3 | 1 |
| CT only | 0 | 1 | 0 |
| TAM + MPA | 0 | 1 | 0 |
| TAM + decadurabolin | 0 | 0 | 1 |

CT, chemotherapy; TAM, tamoxifen; MPA, medroxyprogesterone acetate; 4OH, 4-hydroxyandrostenedione.

for response: three had no measurable or evaluable disease, two received trial treatment for less than 27 days owing to disease progression, one stopped trial treatment after 6 days owing to disease progression, one was lost to follow-up and one had hypercalcaemia the day after the start of the trial treatment (the was treated with clodronate and withdrawn from the trial). Thus there were 72 evaluable patients. Of these, sixteen patients were in retrospect not eligible. One relapsed on adjuvant tamoxifen given for less than 6 months, two were premenopausal, in one the menopausal status was unknown., one was ER negative, two received previous hormonal therapy other than tamoxifen, in one the PS (WHO) was grade 3, four received concomitant endocrine treatment (prednisolone in three, thyroxine in one), three received concomitant biphosphonate treatment and one presented with endocrine disorder (diabetes mellitus).

### *Toxicity*

Thirty-six adverse experiences from 21 patients (26%) were considered to be related to CGS 16949A, the most common of which was mild to moderate nausea in 11 patients (15%). The most frequent experiences are reported in Table II. Two patients were withdrawn from trial treatment owing to poor tolerance: one because of depression and the other because of lethargy. No serious adverse experiences were reported.

There was no statistically significant effect on either plasma sodium or potassium levels at 1 month and 3 months. Minor changes occurred, which were reflected in the sodium–potassium ratio with a significant difference between the 1 and 3 month means. Nevertheless, these differences were not consistent across the dose groups. None of these small effects on sodium, potassium and the sodium–potassium ratio were clinically relevant.

Similarly, there was no significant change in cortisol and aldosterone levels for all these dose groups.

### *Response*

Among clinically evaluable patients (72 patients), 12 patients (17%) had partial responses (PRs) (95% CI 8.9–27.3%), with no complete responders. Fifteen patients (21%) had stable disease (NC) and 45 patients (63%) had progressive disease (PD). Response by treatment regimen is shown in Table III. The median duration of objective response was 36 weeks (calculated from the first day of treatment until the time of diagnosis of disease progression). The data have not been presented by dose group because of the small numbers of patients involved. Among clinically evaluable and eligible patients (56 patients), nine patients had PR (95% CI 7.6–28.3%), with no complete responders. Fourteen patients had NC (25%) and 34 patients (61%) had PD.

**Table II** Fadrozole toxicity

| | *Grade 1* | *Grade 2* | *Grade 3* |
|---|---|---|---|
| Nausea/vomiting | 5 | 6 | 0 |
| State of consciousness (somnolence) | 3 | 0 | 1 |
| Constipation | 2 | 0 | 0 |
| Depression | 0 | 0 | 1 |
| Hot flushes | 4 | 0 | 0 |

**Table III** Response to fadrozole

| | *Fadrozole dose* *1 mg day$^{-1}$* | *2 mg day$^{-1}$* | *4 mg day$^{-1}$* | *Total* |
|---|---|---|---|---|
| PR | 3 (13) | 4 (17) | 5 (20) | 12 (17) |
| NC | 2 (9) | 9 (38) | 4 (16) | 15 (21) |
| PD | 18 (78) | 11 (46) | 16 (64) | 45 (62) |
| Total | 23 | 24 | 25 | 72 |

Data indicate number of patients (%).

### *Time to treatment failure (TTF)*

TTF was calculated in days from the first day of treatment to the date of withdrawal from the trial for any reason (e.g. disease progression, death, lack of tolerance to treatment).

The median TTF was 12.7 weeks. The log-rank test showed no statistically significant differences between the dosage groups. The recording of overall survival was not a trial objective.

### Discussion

This study has shown that fadrozole is a clinically active aromatase inhibitor with a response rate of 17% (95% CI 9–27%) in previously treated patients. This confirms experience in two similar studies with response rates respectively of 23% (95% CI 12–34%) and 16% (95% CI 12–20%) (Raats *et al.*, 1992; Hoeffken *et al.*, 1992). For second-line endocrine therapy these results may be slightly lower than for some other published reports on aromatase inhibitors, including aminoglutethimide (Powles, 1983; Stuart-Harris and Smith, 1984*b*; Miller, 1989) and 4-hydroxyandrostenedione (Goss and Gwyn, 1994). The confidence intervals, however, in this study and in the other two (Raats *et al.*, 1992; Hoeffken *et al.*, 1992) are wide, ranging from 9% to 34%. In addition, a comparison of response rates from second-line hormonal therapies used in different studies does not allow conclusions about the 'best second-line hormonal agent' because of possible selection bias, including the number of non-responders to first-line endocrine therapy (Powles, 1983). Comparative phase III trials are required to answer this question and these are under way. Moreover, in a trial comparing fadrozole with tamoxifen as first-line treatment (Thürlimann *et al.*, 1995) no statistically significant difference in terms of response rate emerged between these two agents (16% and 24% respectively). However, the median time to failure was shorter with fadrozole compared with tamoxifen (4.9 months *vs* 8.3 months), but not statistically significant ($P=0.10$).

The response rates in patients receiving 0.5 mg, 1 mg and 2 mg of fadrozole twice daily were respectively 13.1%, 16.7% and 20.0%. The design of our study and patient numbers involved do not allow dose–response conclusions to be drawn. Raats *et al.* (1992) conducted a study in which the patients were randomised to receive fadrozole 0.5 mg b.d. or 2 mg b.d. The study was designed in order to detect a 30% difference in response rate between the two regimens with a power of 80%; no significant difference was found.

This study has also confirmed that fadrozole is a well-tolerated agent with a low incidence of side-effects. The small number of side-effects reported (nausea, somnolence, hot flushes) were of low WHO grade and a causal relationship with fadrozole was often uncertain. Two patients stopped therapy because of side-effects (somnolence, depression) but again a causal relationship remained uncertain. These side-effects did not appear to be dose related. Others have also reported the low incidence of side-effects with fadrozole (Raats *et al.*, 1992; Hoeffken *et al.*, 1992) and this contrasts favourably with past experience with aminoglutethimide side-effects (Stuart-Harris and Smith, 1984*b*).

In conclusion, fadrozole is a clinically active aromatase inhibitor with a low incidence of side-effects.

### References

BERETTA KR, HOEFFKEN K, KVINNSLAND S, TRUNET P, CHAUDRI HA, BHATNAGAR AS, GOLDHIRSCH A AND CAVALLI F. (1990). CGS 16949A, a new aromatase inhibitor in the treatment of breast cancer – a phase I study. *Ann. Oncol.*, **1,** 421–426.

DOWSETT M. (1990). Clinical development of aromatase inhibitors for the treatment of breast and prostate cancer. *J. Steroid Biochem.*, **37,** 1037–1041.

DOWSETT M, STEIN RC, MEHTA A AND COOMBES RC. (1990). Potency and selectivity of the non-steroidal aromatase inhibitor CGS 16949A in postmenopausal breast cancer patients. *Clin. Endocrinol.*, **32,** 623–634.

DOWSETT M, SMITHERS D, MOORE J, TRUNET P, COOMBES RC, POWLES TJ, RUBENS R AND SMITH IE. (1994). Endocrine changes with the aromatase inhibitor fadrozole hydrochloride in breast cancer. *Eur. J. Cancer*, **30A,** 1453–1458.

GEORGE SL. (1988). The required size and length of a phase III clinical trial. In *Cancer Clinical Trials – Methods and Practice*, Buyse ME, Staquet MJ and Sylvester JR. (eds) Chapter 18. Oxford Medical Publication: Oxford.

GOSS PE AND GWYN KMEH. (1994). Current persectives on aromatase inhibitors in breast cancer. *J. Clin. Oncol.*, **12,** 2460–2470.

HAYWARD JL, RUBENS RD AND CARBONE PP. (1977). Assessment of response to therapy in advanced breast cancer. *Br. J. Cancer.*, **35,** 292–298.

HOEFFKEN K, CHACON R, DOMBERNOWSKY P, GOSS P, WILLEMSE P, ENGAN T, BONTE J, TUBIANA-HULIN M, LOPEZ-LOPEZ JJ, ELOMAA I, CHAUDRI H AND TRUNET P. (1992). Fadrozole hydrochloride (CGS 16949A), a double-blind dose finding study in postmenopausal women with advanced breast cancer. *Ann. Oncol.*, **3,** (suppl.5), 76.

LIPTON A, SANTNER SJ, SANTEN RJ, HARVEY HA, FEIL PD, WHITE-HERSHEY D, BARTHOLOMEW MJ AND ANTLE CE. (1987). Aromatase activity in primary and metastatic human breast cancer. *Cancer*, **59,** 779–782.

LIPTON A, HARVEY HA, DEMERS LM, HANAGAN JR, MULAGHA MT, KOCHAK GM, FIZSIMMONS S, SANDERS SI, SANTEN RJ. (1990). A phase I trial of CGS 16949A – a new aromatase inhibitor. *Cancer*, **65,** 1279–1285.

LONGCOPE C. (1982). Methods and results of aromatization studies in vivo. *Cancer Res.*, **42,** (suppl.), 3307–3311.

LONGCOPE C, PRATT JH, SCHNEIDER SH AND FINEBERG SE. (1978). Aromatization of androgens by muscle and adipose tissue in vivo. *J. Clin. Endocrinol. Metab.*, **46,** 146–152.

MACNEILL FA, JONES AL, JACOBS S, LONNING PE, POWLES TJ AND DOWSETT M. (1992). The influence of aminoglutethimide and its analogue rogletimide on peripheral aromatisation in breast cancer. *Br. J. Cancer*, **66,** 692–697.

MILLER WR. (1989). Aromatase inhibitors in the treatment of advanced breast cancer. *Cancer Treat. Rev.*, **16,** 83–93.

MURRAY R AND PITT P. (1985). Low-dose aminoglutethimide without steroid replacement in the treatment of postmenopausal women with advanced breast cancer. *Eur. J. Cancer Clin. Oncol.*, **21,** 19–22.

POWLES TJ. (1983). The role of aromatase inhibitors in breast cancer. *Semin. Oncol.*, **10,** (suppl.4), 20–24.

RAATS JI, FALKSON G AND FALKSON HC. (1992). A study of fadrozole, a new aromatase inhibitor, in postmenopausal women with advanced metastatic breast cancer. *J. Clin. Oncol.*, **10,** 111–116.

SANTEN RJ, LIPTON A AND KENDALL J. (1974). Successful medical adrenalectomy with aminoglutethimide. Role of altered drug metabolism. *JAMA*, **261,** 1661–1665.

SANTEN RJ, SANTNER S, DAVIS B, VELDHUIS J, SAMOJLIK E AND RUBY E. (1978). Aminoglutethimide inhibits extraglandular oestrogen production in postmenopausal women with breast carcinoma. *J. Clin. Metab.*, **47,** 1257–1265.

SANTEN RJ, DEMERS LM, ADLERCREUTZ H, HARVEY H, SANTNER S, SANDERS S AND LIPTON A. (1989). Inhibition of aromatase with CGS 16949A in postmenopausal women. *J. Clin. Endocrinol. Metab.*, **68,** 99–106.

STEELE RE, MELLOR LB, SAWYER WK, WASVARY JM AND BROWNE LJ. (1987). In vitro and in vivo studies demonstrating potent and selective oestrogen inhibition with the non-steroidal aromatase inhibitor CGS 16949A. *Steroids*, **50,** 147–161.

STEIN RC, DOWSETT M, DAVENPORT J, HEDLEY A, FORD HT, GAZET J-C AND COOMBES RC. (1990). Preliminary study of the treatment of advanced breast cancer in postmenopausal women with the aromatase inhibitor CGS 16949A. *Cancer Res.*, **50,** 1381–1384.

STUART-HARRIS R AND SMITH IE. (1984*a*). Low-dose aminoglutethimide in treatment of advanced breast cancer. *Lancet*, **2,** 604–607.

STUART-HARRIS RC AND SMITH IE. (1984*b*). Aminoglutethimide in the treatment of advanced breast cancer. *Cancer Treat. Rev.*, **11,** 189–204.

THÜRLIMANN B, BERETTA K, BACCHI M, GOLDHIRSCH A, JUNGI WF, CASTIGLIONE M, CAVALLI F, SENN HJ AND FEY M. (1995). First line fadrozole HCl (CGS 16949A) versus tamoxifen (TAM) in advanced breast cancer: prospective randomised study SAKK 20/88. *ASCO Abstracts*, **90,** 98.

WHO. (1979). *WHO Handbook for Reporting Results of Cancer Treatment*. WHO offset publication no. 48. World Health Organization: Geneva.